# HIV-1 Tat-Mediated Human Müller Glial Cell Senescence Involves Endoplasmic Reticulum Stress and Dysregulated Autophagy

**DOI:** 10.3390/v16060903

**Published:** 2024-06-03

**Authors:** Uma Maheswari Deshetty, Nivedita Chatterjee, Shilpa Buch, Palsamy Periyasamy

**Affiliations:** 1 Department of Pharmacology and Experimental Neuroscience, University of Nebraska Medical Center, Omaha, NE 68198-5880, USA; udeshetty@unmc.edu; 2 Vision Research Foundation, Sankara Netralaya, 18, College Road, Chennai 600006, India; drnc@snmail.org

**Keywords:** HIV-1 Tat, senescence, ER stress, autophagy, Müller glial cells

## Abstract

Antiretroviral treatments have notably extended the lives of individuals with HIV and reduced the occurrence of comorbidities, including ocular manifestations. The involvement of endoplasmic reticulum (ER) stress in HIV-1 pathogenesis raises questions about its correlation with cellular senescence or its role in initiating senescent traits. This study investigated how ER stress and dysregulated autophagy impact cellular senescence triggered by HIV-1 Tat in the MIO-M1 cell line (human Müller glial cells). Cells exposed to HIV-1 Tat exhibited increased vimentin expression combined with markers of ER stress (BiP, p-eIF2α), autophagy (LC3, Beclin-1, p62), and the senescence marker p21 compared to control cells. Western blotting and staining techniques like SA-β-gal were employed to examine these markers. Additionally, treatments with ER stress inhibitor 4-PBA before HIV-1 Tat exposure led to a decreased expression of ER stress, senescence, and autophagy markers. Conversely, pre-treatment with the autophagy inhibitor 3-MA resulted in reduced autophagy and senescence markers but did not alter ER stress markers compared to control cells. The findings suggest a link between ER stress, dysregulated autophagy, and the initiation of a senescence phenotype in MIO-M1 cells induced by HIV-1 Tat exposure.

## 1. Introduction

Infection with the human immunodeficiency virus (HIV) leads to the development of acquired immune deficiency syndrome (AIDS), a multisystemic disease that affects several organs, including the eyes [1]. According to the World Health Organization, HIV infection has been recorded in over 50 million individuals worldwide since the 1980s, leading to a global prevalence rate of 0.8% [2]. Every day, nearly 15,000 to 20,000 new HIV infection cases emerge, and the disease burden is disproportionately high in countries with low and middle incomes [3]. Ocular manifestations are among the significant comorbidities of HIV infection, and it is estimated that around three-fourths of HIV-infected patients are prone to ocular diseases [2]. Several studies show that 5–25% of HIV-infected individuals in developing nations may experience visual loss in their lifespan due to ocular complications [1,2].

Choroid and retinal diseases commonly occur in HIV-infected individuals and can eventually lead to blindness [4,5]. In developing countries, HIV-infected patients are prone to opportunistic infections such as herpes zoster ophthalmicus, tuberculosis, papillomavirus-related squamous cell tumors, and toxoplasma [1]. These infections may cause severe visual impairment or blindness, especially when left untreated [1,4,5]. Furthermore, HIV-infected individuals are at an increased risk of ocular damage due to various factors, including vascular abnormalities, opportunistic infections like cytomegalovirus (CMV) retinitis, neuro-ophthalmic disorders, neoplasms, or adverse events from medications [4,5,6]. Among these, CMV retinitis, a consequence of a significant reduction in CD4^+^ T-cell count, is among the leading causes of visual impairment in HIV-infected individuals [5]. Despite the availability of combined antiretroviral therapy (cART), which has significantly improved the lifespan of HIV-infected individuals, ocular complications remain a significant burden for this population [1]. The use of cART has led to changes in the disease presentation and progression of opportunistic infections, such as CMV retinitis, but new ocular complications have emerged, such as immune recovery uveitis [7]. This condition results from an inflammatory response in the eye following the initiation of cART, which can lead to vision loss if left untreated [7].

Recent research has shown that HIV-infected patients are at an elevated risk of age-related non-AIDS mortality and morbidity when compared to HIV-seronegative people [8,9,10,11]. These comorbidities, typically related to the normal aging process, tend to develop earlier in patients with HIV infection than in their HIV-seronegative counterparts [10]. It is believed that individuals with HIV infection may undergo accelerated biological aging in addition to chronological aging, possibly due to immune dysfunction and increased inflammation [8]. Additionally, HIV-infected individuals with AIDS are at an increased risk of developing mid-stage age-associated macular degeneration (AMD) compared to age-matched HIV-seronegative individuals [12]. AMD is a degenerative disease affecting the macula, a retinal part critical for central vision. Intermediate-stage AMD can lead to irreversible vision loss if left untreated [12].

Müller glial cells are the predominant glial cells in the retina, playing crucial roles in maintaining retinal homeostasis and supporting neuronal function. These cells aid in the survival of photoreceptors and neurons, contribute to the structural stability of the retina, and regulate immune and inflammatory responses. They can be activated by various pathological stimuli. While initially neuroprotective, activated Müller glial cells can transition to a state where they no longer support neurons and may instead contribute to neuronal degeneration. Müller glial cells respond to retinal injury by undergoing reactive gliosis, which generally mediates neuroprotective effects. However, persistent gliosis can lead to gliotic scars and contribute to retinal degeneration [13]. Additionally, Müller glial cells are implicated in the initiation of retinal inflammation in diabetic neuropathy, diabetic macular oedema, and AMD [14,15,16]. HIV can infect retinal cells, including Müller glial cells, leading to retinal dysfunction, and potentially causing HIV-related retinal diseases, such as HIV retinopathy. HIV-related retinopathies can result in visual impairment and decreased survival, necessitating lifelong and challenging maintenance therapy [17].

Various neurological diseases, including brain trauma ischemia, Parkinson’s, and Alzheimer’s disease, have demonstrated the implications of endoplasmic reticulum (ER) stress [18,19,20]. The ER regulates vital cellular functions, like protein synthesis and folding, calcium storage, and lipid biosynthesis. The disruption of ER homeostasis due to various physiological and pathological stimuli may result in the aggregation of misfolded proteins, inducing ER stress. The process of an unfolded protein response (UPR), regulated by three major pathways, protein kinase RNA-like ER kinase (PERK), activating transcription factor 6 (ATF6), or inositol-requiring enzyme 1α (IRE1α) is activated to alleviate ER stress. The UPR enhances cell survivability by decreasing protein translation and inducing chaperone expression, endogenous antioxidant responses, and, eventually, proteins undergoing ER-associated degradation and autophagy. However, the prolonged activation of UPR can also lead to apoptosis [21]. It is important to note that adapting to ER stress through UPR and the appropriate regulation of closely linked autophagy plays a significant role in cell homeostasis [22]. Recent studies have reported the accumulation of amyloid beta (Aβ) in the brains of HIV-infected individuals, suggesting the possible association with protein misfolding and initiation of the UPR in the pathogenesis of HIV infection [23,24].

Furthermore, senescence is a complex cell phenomenon due to different stress types, such as oxidative stress, telomere shortening, oncogene activation, and DNA damage. As senescence is a stress response, examining whether ER stress occurs simultaneously with cellular senescence and plays a role in initiating or sustaining it is crucial [25]. This study investigates how ER stress and impaired autophagy contribute to cellular senescence induced by HIV-1 Tat in MIO-M1 cells. Understanding the function of ER stress in HIV-1 infection and cellular senescence is critical for developing successful treatment approaches. HIV-1 infection is linked with various neurological disorders, and the contribution of ER stress in their development is becoming increasingly evident. However, the exact mechanisms by which ER stress leads to the development and progression of HIV-related neurological disorders are not entirely understood. Similarly, while cellular senescence has been linked with the development and progression of various age-related diseases, the relation between ER stress and cellular senescence in HIV-1 infection is still mostly unexplored. Therefore, investigating the function of ER stress and autophagy in the context of HIV-1-induced cellular senescence could provide vital information on HIV-associated neurological disorder pathogenesis and potentially lead to the development of novel therapeutic strategies.

## 2. Materials and Methods

### 2.1. Chemicals and Reagents

HIV-1 recombinant Tat (Cat. No. 1032–10) was procured from ImmunoDX, LLC (Woburn, MA, USA). Sodium 4-phenylbutyrate (4-PBA, Cat. No. 567616) was purchased from EMD Millipore Corporation (Burlington, MA, USA), whereas 3-MA (Cat. No. M9281), bafilomycin A1 (Cat. No. B1793), and rapamycin (Cat. No. R0395) were obtained from Sigma-Aldrich (St. Louis, MO, USA). Antibodies such as BiP (BD Biosciences, Franklin Lakes, NJ, USA, Cat. No. 610979), vimentin (Cat. No. 5741T), beclin-1 (Santa Cruz Biotechnology, Inc., Dallas, TX, USA, Cat. No. sc-11427), p62 (MBL International, Schaumburg, IL, USA, Cat. No. PM045), LC3 (Novus Biological Company, Centennial, CO, USA, Cat. No. NB100-2220), P21 (Abcam, Boston, MA, USA, Cat. No. ab109199), Peroxidase-AffiniPure Goat Anti-Rabbit IgG (H + L) (Jackson ImmunoResearch Inc., West Grove, PA, USA, Cat. No. 111035-003) and Peroxidase-conjugated AffiniPure Goat Anti-Mouse IgG (H + L) (Jackson ImmunoResearch Inc. West Grove, PA, USA, Cat. No. 115-035-003) were procured as mentioned from the commercial vendors.

### 2.2. Cell Culture and Treatments

The human MIO-M1 cells were cultured in 6-well culture plates (0.2 × 10^6^ cells/well density) using Dulbecco’s modified Eagle’s medium (Corning, Oneonta, NY, USA, Cat. No. 10-013-CV) comprising penicillin-streptomycin (Life Technologies, Carlsbad, CA, USA, Cat. No. 15140-122), and 10% fetal bovine serum—Premium, heat-inactivated (Atlanta Biologicals, Flowery Branch, GA, USA, Cat. No. S11150H). MIO-M1 cells were serum-starved overnight before treatments. For dose curve experiments, MIO-M1 cells were exposed to HIV-1 Tat with different doses (25 to 200 ng/mL) for 24 h. For time course experiments, MIO-M1 cells were exposed to a selected dose of 50 ng/mL for 3 to 48 h. The dosage and duration of HIV-1 Tat exposure for MIO-M1 cells were established based on the expression of BiP and vimentin protein levels and thus selected as 50 ng/mL and 24 h, respectively, for further experiments.

### 2.3. Western Blotting

After completing various treatments, MIO-M1 cells were collected and lysed with RIPA buffer consisting of phosphatase cocktail inhibitor (Thermo Fisher Scientific, Pittsburgh, PA, USA, Cat. No. 78426) and protease cocktail inhibitor (Thermo Fisher Scientific, Pittsburgh, PA, USA, Cat. No. 78429). The protein lysates were prepared as per standard protocols and reported previously [26,27,28,29,30,31]. The expression of proteins like BiP, vimentin, Beclin1, LC3B-II, p62, P21, and IL1β were determined by Western blotting.

### 2.4. LC3 Accumulation and p62 Degradation Assays

LC3 accumulation and p62 degradation assays were performed as per standard protocols and reported previously [27,32]. Briefly, MIO-M1 cells were exposed to HIV-1 Tat (50 ng/mL), and four hours before harvesting, they were further treated with a saturating concentration (400 nM) of bafilomycin A1 (BAF). Subsequently, the cells were harvested and processed to evaluate the expression of LC3 and p62.

### 2.5. Immunocytochemistry

Immunocytochemistry was performed as per standard protocol with minor modifications [26,27,28,29,30,32]. After treatment, MIO-M1 cells were briefly rinsed with 1× PBS, followed by fixation with 4% paraformaldehyde at room temperature for 20 min. After fixation, 0.3% Triton X-100 (Fisher Scientific, Hampton, NH, USA, Cat. No. BP151-500) in PBS was used to permeabilize the cells, followed by blocking using bovine serum albumin at room temperature for 1 h. Subsequently, MIO-M1 cells were incubated with the appropriate primary antibody (diluted 1:200) overnight at 4 °C. After the initial antibody incubation, cells were exposed to secondary antibodies for 2 h. These included Alexa Fluor 594-conjugated goat anti-chicken IgG (H + L) and Alexa Fluor 488-conjugated goat anti-mouse IgG (H + L) to identify the presence of the specified proteins. The coverslips were subsequently mounted using ProLong Gold Antifade Reagent with DAPI (Thermo Fisher Scientific, Inc. Pittsburgh, PA, USA, Cat. No. P36935) for further imaging.

### 2.6. Evaluation of Autophagosome Formation and Maturation

Autophagosome formation and maturation assay was performed as per standard protocol with minor modifications [27,32]. Briefly, MIO-M1 cells were transfected with the tandem fluorescent-tagged LC3B plasmid (ptfLC3; Addgene, Watertown, MA, USA, Cat. No. 21074), and the transfected cells were exposed to HIV-1 Tat (50 ng/mL), and autophagy inducer rapamycin (100 nM) for 24 h. Additionally, at a concentration of 400 nM, BAF was added 4 h before the 24 h incubation period. Then, the cells were fixed for imaging to analyze these images for the accumulation of autophagosomes and autolysosomes.

### 2.7. Senescence-Associated (SA) β-Galactosidase (Gal) Staining Assay

This assay was performed as per standard protocol with minor modifications [33]. Briefly, MIO-M1 cells were exposed to HIV-1 Tat (50 ng/mL) along with suitable controls for 24 h. After that, the cells were fixed on a coverslip using 4% paraformaldehyde for SA β-gal staining. Images were then captured and analyzed for blue positive and negative cells using ImageJ software (version 1.54i). At least 10 images per treatment were analyzed, and ~200 cells were counted per image in triplicate samples.

### 2.8. Statistical Analysis

The data are represented as mean ± standard error of the mean (SEM). GraphPad Prism software version 10.1 (GraphPad Software, Inc. San Diego, CA, USA) was used to perform the statistical analysis. The Kruskal–Wallis non-parametric test was used to assess the statistical significance between groups. If the analysis of variance yielded significant results, post hoc testing was conducted using Dunn’s multiple comparison test for inter-group comparisons (between 2 groups). In this study, *p* < 0.05 was considered statistically significant.

## 3. Results

### 3.1. HIV-1 Tat Induces ER Stress and Autophagy in MIO-M1 Cells

**Figure 1:**
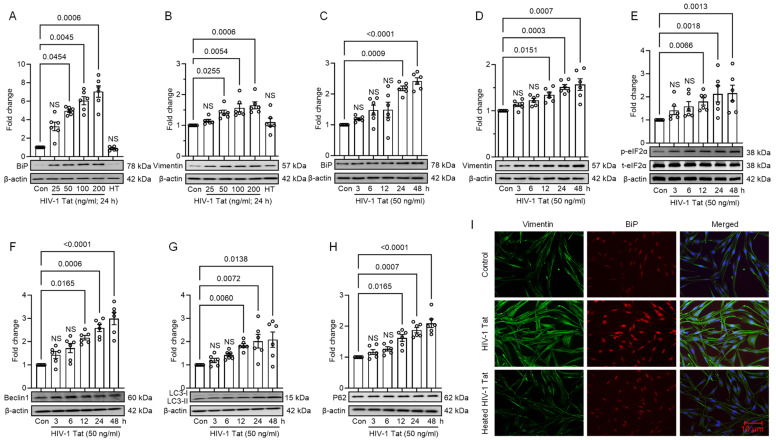
HIV-1 Tat initiates ER stress and autophagy in MIO-M1 cells. Representative Western blots and bar graphs show the HIV-1 Tat-mediated dose-dependent upregulation of (**A**) BiP (ER stress marker) and (**B**) vimentin (activation marker) in MIO-M1 cells. Representative Western blots and bar graphs demonstrate the HIV-1 Tat-mediated time-dependent upregulation of (**C**) BiP, (**D**) vimentin, (**E**) p-eIF2α, (**F**) Beclin1, (**G**) LC3B, and (**H**) p62 in MIO-M1 cells. (**I**) Representative immunofluorescence images depict the co-localized staining of BiP and vimentin in MIO-M1 cells. The data are represented as mean ± SEM from 6 independent experiments. The dot or small circle in each bar graph represents a single data point. Nonparametric Kruskal–Wallis one-way ANOVA followed by the Dunn multiple comparison test was used to determine the statistical significance between groups; *p* < 0.05 vs. control.

To investigate the potential of HIV-1 Tat to trigger ER stress, we exposed the MIO-M1 cells to different doses of HIV-1 Tat (25, 50, 100, and 200 ng/mL) for 24 h. We then assessed the expression levels of the ER stress marker, BiP (binding immunoglobulin protein), and the glial cell-specific marker, vimentin, using Western blotting. Exposure of MIO-M1 cells to HIV-1 Tat resulted in a dose-dependent upregulation in the expression levels of BiP and vimentin (Figure 1A,B). Notably, at a concentration of 50 ng/mL, HIV-1 Tat significantly (*p* < 0.05) increased the protein expression of both BiP and vimentin (Figure 1A,B). Thus, 50 ng/mL of HIV-1 Tat was chosen for subsequent experiments. Recent studies have reported that the levels of HIV-1 Tat in serum range from 0 to 14 ng/mL in HIV-infected individuals. Furthermore, only 25.4% of HIV-1-infected individuals exhibited HIV-1 Tat concentrations above the established cut-off value (>2.5 ng/mL), with a median concentration of 4.518 ng/mL [34]. Another study detected HIV-1 Tat in the cerebrospinal fluid of well-suppressed patients on cART at concentrations ranging from 0.20 ng/mL to 6.5 ng/mL [35]. Despite these findings, a concentration of 50 ng/mL for HIV-1 Tat protein is commonly employed in research as it closely approximates the range of 2–40 ng/mL observed in the plasma samples of HIV-infected patients [36,37,38,39]. At this concentration, HIV-1 Tat induces various cellular responses pertinent to HIV-1 pathogenesis without causing excessive cell death. Specifically, it activates transcription, promotes viral replication, and triggers inflammatory pathways, all of which are essential for studying the effects of HIV-1 on host cells [40,41,42,43].

Next, we conducted time-course experiments to examine the optimal time for inducing ER stress and autophagy. MIO-M1 cells were exposed to HIV-1 Tat (50 ng/mL) for various time intervals (0, 3, 6, 12, 24, and 48 h), and the expression levels of the ER stress markers (BiP, phosphorylated eIF2α) and autophagy markers (LC3B, Beclin1, and p62) were determined utilizing Western blotting. The results demonstrated that exposure of MIO-M1 cells to HIV-1 Tat caused a significant (*p* < 0.05) upregulation in the levels of BiP, phosphorylated eIF2α, LC3B, Beclin1, and p62, starting from 6 h onwards (Figure 1C–H). Moreover, this increase was sustained for up to 48 h, indicating prolonged activation of ER stress and autophagy in the context of HIV-1 Tat exposure (Figure 1C–H). To further evaluate the HIV-1 Tat effects on MIO-M1 cells, we performed immunofluorescence staining to study the expression of BiP and vimentin using specific primary antibodies. Control MIO-M1 cells displayed a normal morphology with basal levels of BiP and vimentin expression. However, MIO-M1 cells exposed to HIV-1 Tat (50 ng/mL, 24 h) showed a significant increase in both BiP and vimentin expression, indicating altered cellular morphology (Figure 1I). In contrast, cells treated with heat-inactivated HIV-1 Tat showed no significant changes in cellular morphology, confirming that the observed effects were specific to active HIV-1 Tat (Figure 1I).

### 3.2. HIV-1 Tat-Mediated Activation of ER Stress Results in Dysregulated Autophagy in MIO-M1 Cells

As evident from our previous results, the upregulation of p62 expression in MIO-M1 cells treated with HIV-1 Tat raised suspicions of dysregulated autophagy. Generally, autophagy induction leads to the downregulation of p62 protein levels. To investigate this phenomenon further and determine the involvement of dysregulated autophagy in the present context, we conducted several assays related to autophagic flux in MIO-M1 cells. We employed the LC3 accumulation assay, p62 degradation assay, and RFP-GFP-LC3 overexpression assay to assess autophagic flux. Dysfunctional fusion between autophagosomes and lysosomes can reduce autophagic clearance, resulting in increased cellular toxicity. The conversion of LC3-I to LC3-II via lipidation occurs during the formation of autophagosomes, and LC3-II associates selectively with fully formed autophagosomes. Therefore, the abundance of LC3-II directly reflects the presence of autophagosomes. The autophagic flux assay evaluates LC3-II accumulation when BAF inhibits autophagosome-lysosome fusion.

**Figure 2:**
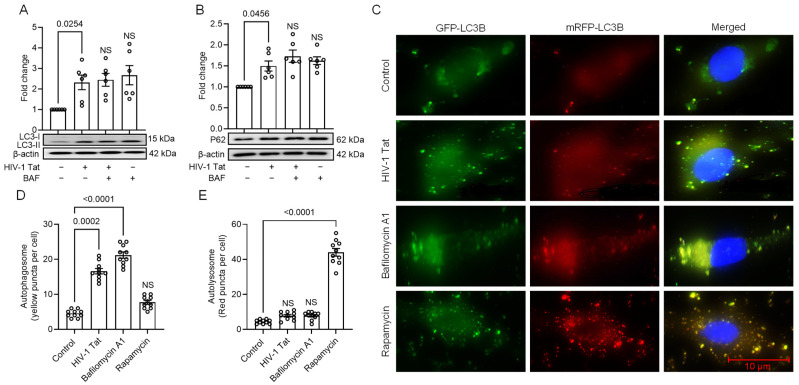
HIV-1 Tat-mediated activation of ER stress causes dysregulated autophagy in MIO-M1 cells. (**A**,**B**) Representative Western blots show the expression of (**A**) LC3B-I, LC3B-II, and (**B**) p62 in MIO-M1 cells exposed to HIV-1 Tat (50 ng/mL) for 24 h followed by treatment with 400 nM BAF, added 4 h prior to the 24 h treatment period. The β-actin was used as a loading control for all experiments. (**C**) MIO-M1 cells transfected with tandem fluorescent-tagged LC3B plasmid followed by HIV-1 Tat (50 ng/mL) and treated with rapamycin (10 nM) for 24 h. Scale bar: 10 μm. Bar graph depicts the number of (**D**) autophagosomes and (**E**) autolysosomes in MIO-M1 cells transfected with a tandem fluorescent-tagged LC3B plasmid and treated with HIV-1 Tat and rapamycin for 24 h, showing the number of puncta per cell across 10 images per treatment group. The data are represented as mean ± SEM from 6 independent experiments. The dot or small circle in each bar graph represents a single data point. Nonparametric Kruskal–Wallis one-way ANOVA followed by the Dunn multiple comparison test was used to determine the statistical significance between multiple groups, *p* < 0.05.

In our study, MIO-M1 cells were treated with HIV-1 Tat (50 ng/mL) for 24 h and treated with or without a saturating concentration of BAF (400 nM) for 4 h before harvesting the cells. As depicted in Figure 2, no significant differences were observed in the accumulation of LC3-II in MIO-M1 cells treated with HIV-1 Tat, regardless of the presence or absence of BAF (Figure 2A). This suggests that HIV-1 Tat-induced autophagy activation may not be efficiently processed and degraded in lysosomes, indicative of dysregulated autophagy. Moreover, we also conducted a p62 degradation assay, which showed similar results to the LC3-II assay. p62 is a crucial adaptor protein that binds to ubiquitin, facilitating the recruitment of LC3 and leading to autophagosome formation and the selective degradation of autophagic cargoes. The accumulation of p62 protein and impaired degradation are associated with the degradation rate of autophagic vesicles. In MIO-M1 cells treated with HIV-1 Tat, we found no significant alteration in the accumulation of p62, irrespective of the absence or presence of BAF (Figure 2B).

To further confirm the dysregulation of autophagy flux induced by HIV-1 Tat, we conducted additional experiments using tandem fluorescent-tagged LC3 plasmid. After HIV-1 Tat exposure, red and green fluorescence was emitted by the tandem LC3 plasmid, resulting in merged yellow puncta indicative of mRFP and GFP-LC3 colocalization. However, in the acidic pH environment of lysosomes, the GFP signal associated with the tandem fluorescent-tagged LC3 fusion protein is quenched, while the red fluorescence persists and is observed as red puncta. Consequently, an enhanced yellow puncta accumulation signifies impaired autophagy, whereas an increased red punctum accumulation indicates enhanced autophagy flux. Remarkably, the transfection of MIO-M1 cells with the tandem fluorescent-tagged LC3 reporter plasmid and subsequent exposure to HIV-1 Tat (50 ng/mL, 24 h) displayed a significant upregulation in yellow puncta and a simultaneous reduction in red puncta, signifying incomplete maturation of the autophagosome. Similarly, treating MIO-M1 cells with BAF also resulted in an increased number of yellow puncta. In contrast, MIO-M1 cells exposed to rapamycin (autophagy inducer; 100 nM) showed an elevated formation of red puncta (Figure 2C–E), suggesting enhanced autophagic flux.

### 3.3. HIV-1 Tat Activates ER Stress, Autophagy, and Senescence in MIO-M1 Cells

To further explore the implications of HIV-1 Tat-induced ER stress and autophagy in MIO-M1 cells, we investigated whether these cellular responses were associated with developing a senescent phenotype. For this purpose, we conducted Western blot analysis to assess the expression of p21 protein, a well-known senescence marker, in MIO-M1 cells treated with HIV-1 Tat (50 ng/mL) for variable time points.

**Figure 3:**
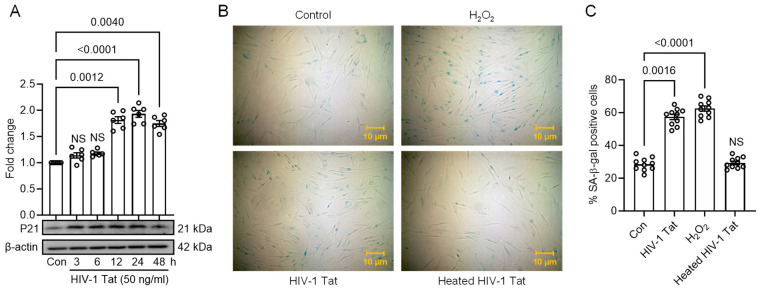
HIV-1 Tat induces senescence in MIO-M1 cells. (**A**) Representative Western blots showing the time-dependent upregulation of expression of p21 (senescence marker) in HIV-1-treated MIO-M1 cells for 24 h. β-actin was probed as a protein-loading control for all experiments. Representative images showing (**B**) the cellular morphology and SA-β-gal (blue cells) staining, and (**C**) quantification of SA-β-gal positive cells on HIV-1 Tat-treated MIO-M1 cells for 24 h. The data are represented as mean ± SEM from 6 independent experiments. The dot or small circle in each bar graph represents a single data point. Nonparametric Kruskal–Wallis one-way ANOVA followed by the Dunn multiple comparison test was used to determine the statistical significance between multiple groups, *p* < 0.05.

As demonstrated in Figure 3A, a time-dependent increase in the p21 protein expression was observed with HIV-1 Tat exposure, indicating a potential link between ER stress, autophagy induction, and senescence. Additionally, MIO-M1 cells were treated with HIV-1 Tat (50 ng/mL, 24 h), followed by SA-β-gal staining to further validate the presence of a senescent phenotype. The results showed a significant increase in blue staining, indicating the presence of senescent cells, in the HIV-1 Tat-treated MIO-M1 cells compared to the control cells. As a positive control, we used H_2_O_2_ (150 μM for 2 h) treatment, which also induced SA-β-gal activity in senescent cells (Figure 3B,C).

### 3.4. HIV-1 Tat-Mediated Senescence Signalling Involves Upstream Activation of ER Stress

**Figure 4:**
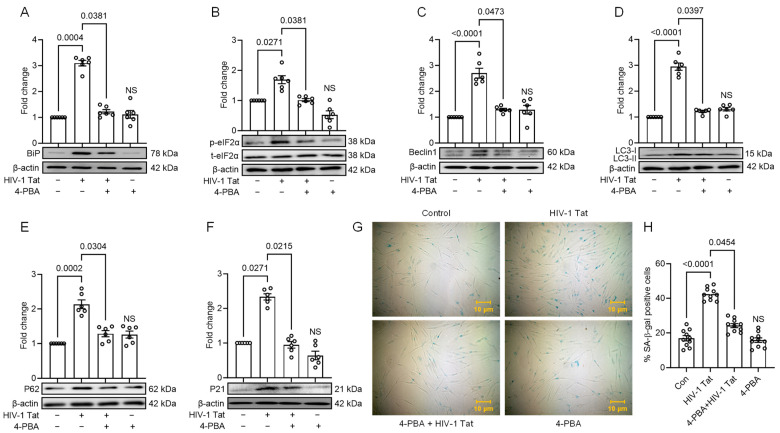
HIV-1 Tat-mediated senescence signaling involves upstream activation of ER stress. Representative Western blots showing the upregulation of expression of ER stress marker- (**A**) BiP, (**B**) p-eIF2α; autophagy markers- (**C**) Beclin1, (**D**) LC3B, and (**E**) p62; and senescence marker- (**F**) p21 in HIV-1- treated MIO-M1 cells for 24 h. However, expression of these markers was downregulated when MIO-M1 cells were pre-treated with 25 μM 4-PBA (ER stress inhibitor). β-actin was probed as a protein loading control for all experiments. Representative images showing (**G**) the cellular morphology and SA-β-gal (blue cells) staining, and (**H**) quantification of SA-β-gal positive cells on 4-PBA pre-treated MIO-M1 cells for 1 h followed by HIV-1 Tat-treatment for 24 h. The data are represented as mean ± SEM from 6 independent experiments. The dot or small circle in each bar graph represents a single data point. Nonparametric Kruskal—Wallis One-way ANOVA followed by the Dunn multiple comparison test was used to determine the statistical significance between multiple groups, *p* < 0.05.

To investigate the relationship between ER stress, autophagy, and senescence in the scenario of HIV-1 Tat exposure, we aimed to confirm whether ER stress activation leads to the subsequent induction of autophagy and senescent phenotype. To achieve this, we pre-treated MIO-M1 cells with 25 mM of 4-PBA, an ER stress inhibitor, for 1 h, followed by treatment with the HIV-1 Tat (50 ng/mL) for 24 h. We then evaluated the expression of ER stress, autophagy, and senescence markers using Western blot analysis. As shown in Figure 4A–F, the results demonstrate that the upregulation of ER stress markers, including BiP and p-eIF2-α, as well as autophagy markers, LC3B, Beclin1, and p62, and the senescence marker p21, induced by HIV-1 Tat, significantly (*p* < 0.05) decreased in MIO-M1 cells that were pre-treated with 4-PBA.

As shown in Figure 4A–F, the results demonstrate that the upregulation of ER stress markers, including BiP and p-eIF2-α, as well as autophagy markers, LC3B, Beclin1, and p62, and the senescence marker p21, induced by HIV-1 Tat, significantly (*p* < 0.05) decreased in MIO-M1 cells that were pre-treated with 4-PBA. These findings were comparable to the observations made in MIO-M1 cells not exposed to HIV-1 Tat, indicating that inhibition of ER stress attenuated the subsequent activation of autophagy and senescence pathways. We performed SA-β-gal staining following the abovementioned treatments to further validate the senescence phenotype. As shown in Figure 4, we observed a decreased SA-β-gal phenotype in MIO-M1 cells that were pre-treated with 4-PBA, as compared to the MIO-M1 cells treated with HIV-1 Tat alone, suggesting that inhibition of ER stress also led to a decrease in the senescent phenotype development (Figure 4G,H).

**Figure 5:**
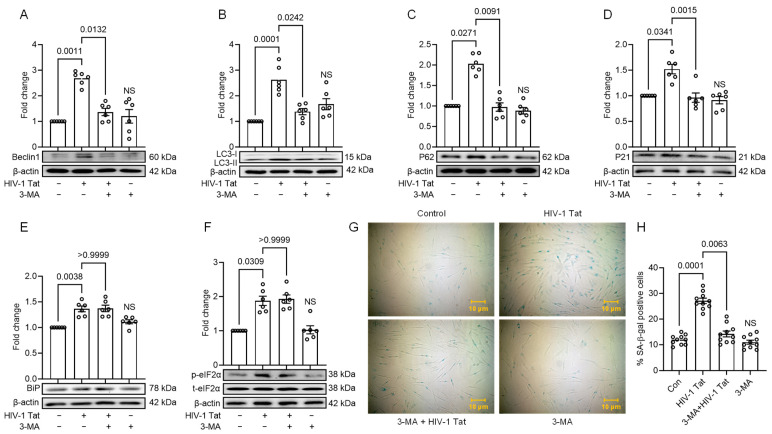
HIV-1 Tat-mediated senescence signaling involves upstream activation of autophagy. Representative Western blots showing the upregulated expression of autophagy markers: (**A**) Beclin1, (**B**) LC3B, and (**C**) p62; and senescence marker: (**D**) p21, and no change in expression of ER stress markers: (**E**) BiP, (**F**) p-eIF2α in HIV-1- treated MIO-M1 cells for 24 h. However, expression of autophagy and senescence markers was downregulated when MIO-M1 cells were pre-treated with 2 μM 3-MA (autophagy inhibitor). β-actin was probed as a protein-loading control for all experiments. Representative images showing the (**G**) cellular morphology and SA-β-gal (blue cells) staining, and (**H**) quantification of SA-β-gal positive cells on 3-MA pre-treated MIO-M1 cells for 1 h followed by HIV-1 Tat-treatment for 24 h. The data are represented as mean ± SEM from 6 independent experiments. The dot or small circle in each bar graph represents a single data point. Nonparametric Kruskal–Wallis one-way ANOVA followed by the Dunn multiple comparison test was used to determine the statistical significance between multiple groups, *p* < 0.05.

We next employed a pharmacological inhibition approach to investigate the role of HIV-1 Tat-induced dysregulated autophagy in initiating senescence and its potential effects on the activation of ER stress. The cells were pre-treated with 2 µM of 3-MA (an autophagy inhibitor) for 1 h, followed by HIV-1 Tat (50 ng/mL) for 24 h. Subsequently, we evaluated the expression of autophagy, senescence, and ER stress markers. As demonstrated in Figure 5A–D, the HIV-1 Tat-induced upregulation of autophagy markers like LC3B, Beclin1, and p62, as well as senescence markers such as p21, was suppressed significantly (*p* < 0.05) in MIO-M1 cells that were pre-treated with 3-MA. These findings were consistent with the observations made in untreated MIO-M1 cells, indicating that the inhibition of autophagy reduced the induction of autophagy and senescence pathways. However, it is noteworthy that no obvious change in the expression of ER stress markers was observed among the treatments (Figure 5E,F) compared to the control MIO-M1 cells. This suggests that the ER stress pathway may be upstream of the autophagy process in the present context and that HIV-1 Tat-mediated autophagy activation does not exert feedback effects on ER stress induction. Following the above approach, we also performed SA-β-gal staining to confirm the senescence phenotype. Strikingly, we observed a reduced SA-β-gal-stained cellular phenotype in cells that were pre-treated with 3-MA compared to the cells treated with HIV-1 Tat alone, supporting the role of autophagy in initiating the senescence process (Figure 5G,H).

## 4. Discussion

The ophthalmic symptoms of HIV infection can be easily missed because they often appear late during AIDS when other symptoms are more severe. Although HAART has reduced the number of cases of some debilitating eye conditions, such as CMV retinitis, it has not eliminated new cases or prevented complications like retinal detachment or immune recovery uveitis [44]. Infection leading to the subsequent stimulation of immune system cells continues to be the predominant factor behind ocular damage. The environment within the eye is characterized by its dual qualities of suppressing immune responses and reducing inflammation [44]. Within the retina, a minority of glial cells are identified as astrocytes that express GFAP, a protein-expression marker of cellular activation. The prevalent cell category is Muller glia. Nevertheless, studies have revealed a strong association between retinal damage and degeneration, marked by intense astrogliosis in the optical nerve and the Muller glial activation [45].

Apart from chemokines, elevated quantities of viral proteins and pro-inflammatory cytokines are recognized as important factors in retinal degeneration [45,46]. Specifically, HIV-1 Tat, a protein released from infected cells in the central nervous system, has been demonstrated to have neurotoxic effects in both cell cultures and animal models of the disease [47,48]. The ability of HIV-1 Tat to stimulate HIV-1 gene expression is well-established, achieved through interactions with elements such as the transactivation responsive element TAR within the HIV-1 long terminal repeat promoter, human cyclin T1, and CDK9. Moreover, HIV-1 Tat has been observed to have multifaceted impacts on host cells, such as the induction of HIV-1 replication, apoptosis, oxidative stress, inflammation, and the release of neurotransmitters [49,50,51,52,53].

Several neurological conditions, encompassing brain injuries, ischemia, Parkinson’s, and Alzheimer’s disease, have exhibited associations with the consequences of ER stress [18,19,20]. However, prolonged UPR activation can also lead to apoptosis [21]. It is important to note that adapting to ER stress through UPR and the appropriate regulation of closely linked autophagy plays an important role in cell homeostasis [22]. Autophagy has the potential to degrade both misfolded proteins and inclusion bodies, offering a possible therapeutic avenue. Autophagy can be categorized into two types: constitutive autophagy, which works continuously to break down abnormal proteins and maintain cellular balance, and inducible autophagy, which is activated on a larger scale in response to various stressors to protect cells from damage [54]. The UPR and autophagy are considered to occur concurrently and play major roles in the pathogenesis of neurodegenerative diseases and cancer. The UPR can trigger autophagy, and in turn, autophagy can mitigate the effects of the UPR [55]. Interestingly, senescence is considered to occur in parallel with autophagy, sharing various pathways and mechanistic regulators; however, both autophagy and senescence are induced by various stressors [56]. For instance, elevated levels of reactive oxygen species (ROS) cause cytotoxicity, leading to mitochondrial dysfunction, genomic instability, and the accumulation of misfolded proteins. To counteract this stress caused by ROS, cells induce autophagy to eliminate and recycle damaged organelles and proteins [57,58,59]. Conversely, oxidative stress can result in significant DNA damage within cells, triggering a senescent cell cycle arrest as the cell attempts to repair the damage [56,60]. Thus, the balance between ER stress, autophagy, and senescence is crucial for maintaining cellular health. A disruption of this balance can contribute to various diseases, including neurodegenerative disorders and cancer. The interplay between these processes is complex and not fully understood, presenting opportunities for therapeutic interventions.

In the present study, a human retinal Muller glial cell line was used to evaluate the effect of HIV-1 Tat protein on the release of the ER stress mediators initiating dysregulated autophagy. Müller glial cells are macroglial retinal cells that play neuroprotective roles but can also contribute to neuronal degeneration [13]. In line with this, we showed that HIV-1 Tat-induced activation of MIO-M1 cells involved modifications in the ER stress pathway and dysregulated autophagy. Our results demonstrated that exposure of MIO-M1 cells to HIV-1 Tat upregulated the expression of the ER chaperone protein BiP and vimentin in both a dose- and time-dependent manner, indicating the initiation of ER stress and activation of these cells. Also, it was observed that on HIV-1 Tat exposure, the activation of ER stress led to the induction of autophagy, as evident by the upregulated expression of LC3B, Beclin1, and p62. However, we speculated and confirmed that ER-stress-initiated autophagy is dysregulated autophagy, as there were no changes observed in the LC3B and p62 expression when the MIO-M1 cells were exposed to a known inhibitor BAF and the increased yellow puncta in the HIV-1 Tat and BAF-treated MIO-M1 cells are in accordance with previous reported studies [61,62,63,64,65,66].

Moreover, senescence represents a multifaceted cellular process brought about by various forms of stress, including oxidative stress, DNA damage, activation of oncogenes, and telomere shortening. Given that senescence is a stress response, we explored whether ER stress occurs concomitantly with cellular senescence and whether it plays a role in initiating or sustaining this process [25]. In this context, we observed a senescence-like phenotype demonstrated by upregulated p21 expression and SA-β-gal blue staining phenotype in the HIV-1 Tat-treated MIO-M1 cell line. We also demonstrated that HIV-1 Tat-induced senescence signaling involves the upstream activation of ER stress, as the expression of ER stress, autophagy, and senescence mediators were downregulated in 4-PBA (ER stress inhibitor)-treated MIO-M1 cells. Interestingly, the ER stress markers remained unchanged, whereas the downregulation of autophagy and senescence mediators was observed in 3-MA (autophagy inhibitor)-treated MIO-M1 cells, indicating that HIV-1 Tat-induced senescence signaling involves the upstream activation of ER stress. Overall, ER stress leads to dysregulated autophagy, which, in turn, regulates senescence.

## 5. Conclusions

In the present study, we showed that the treatment of MIO-M1 cells with HIV-1 Tat increased ER stress and dysregulated autophagy, eventually culminating in the initiation of the senescence phenotype. Therefore, investigating the role of ER stress and autophagy in the context of HIV-1-induced cellular senescence could provide vital insights into the pathogenesis of HIV-associated neurological disorders and potentially lead to the development of novel therapeutic strategies.

## Data Availability

The data that support the findings of this study are available from the corresponding authors upon reasonable request.
